# Translating clinical training into practice in complex mental health systems: Toward opening the 'Black Box' of implementation

**DOI:** 10.1186/1748-5908-3-33

**Published:** 2008-06-03

**Authors:** Greer Sullivan, Dean Blevins, Michael R Kauth

**Affiliations:** 1 South Central Mental Illness Research, Education, and Clinical Center (SC-MIRECC), Central Arkansas Veterans Healthcare System, North Little Rock, USA; 2 Central Arkansas Veterans Healthcare System, HSR&D, Center for Mental Healthcare and Outcomes Research (CeMHOR), North Little Rock, USA; 3 University of Arkansas for Medical Sciences, Department of Psychiatry, Division of Health Services Research, Little Rock, USA; 4 Michael E. DeBakey Veterans Affairs Medical Center, Houston, USA; 5 Menninger Department of Psychiatry & Behavioral Sciences, Baylor College of Medicine, Houston, USA

## Abstract

**Background:**

Implementing clinical training in a complex health care system is challenging. This report describes two successive trainings programs in one Veterans Affairs healthcare network and the lessons we drew from their success and failures. The first training experience led us to appreciate the value of careful implementation planning while the second suggested that use of an external facilitator might be an especially effective implementation component. We also describe a third training intervention in which we expect to more rigorously test our hypothesis regarding the value of external facilitation.

**Results:**

Our experiences appear to be consonant with the implementation model proposed by Fixsen. In this paper we offer a modified version of the Fixsen model with separate components related to training and implementation.

**Conclusion:**

This report further reinforces what others have noted, namely that educational interventions intended to change clinical practice should employ a multilevel approach if patients are to truly benefit from new skills gained by clinicians. We utilize an implementation research model to illustrate how the aims of the second intervention were realized and sustained over the 12-month follow-up period, and to suggest directions for future implementation research. The present report attests to the validity of, and contributes to, the emerging literature on implementation research.

## Background

There is an ongoing need within healthcare systems to train clinicians to deliver evidence-based care, particularly when clinicians are well past their initial training. Educational programs may be especially challenging in mental health because adequate training in many therapeutic techniques, such as cognitive behavioral therapy or psychosocial rehabilitation skills, is typically time consuming and labor intensive for both the trainer and trainee. Once clinicians are trained, implementing new practices in treatment settings in complex health systems poses additional challenges [1-3].

In this debate paper, we describe a series of training interventions for mental health providers undertaken by the Department of Veterans Affairs (VA) South Central Mental Illness Research, Education, and Clinical Center (SC MIRECC) in a large, geographically dispersed network of care, Veterans Integrated Service Network 16 (VISN 16). The experience we gained from these interventions has informed our thinking about training and implementation basics. Now we are preparing to test some of the implementation components that we have delineated through our direct experiences.

The first intervention consisted of a basic training experience for clinicians, but its implementation into practice appeared limited by inattention to contextual issues. This experience led us to make a distinction between 'training' and 'implementation' phases of the intervention. The second intervention, designed with greater attention to contextual implementation issues, utilized facilitation to promote both training and implementation phases, and found much wider uptake into clinical practice. Our successful experience with the second intervention prompted further refinement of our intervention facilitation model, and we consequently identified a distinction between internal and external facilitation. Finally, our third intervention will open the 'black box' of intervention by systematically examining the value of external facilitation. To date, most large-scale effectiveness research has compared multi-faceted training and implementation efforts to treatment as usual conditions. Testing specific intervention components like external facilitation will enable us to open the black box of multi-faceted training and implementation and examine the value of specific components.

### Setting

One of the largest VA networks, VISN 16 serves approximately two million veterans across an eight-state region from Oklahoma to the Florida panhandle. Mental health services are coordinated by the network mental health product line manager and the advisory council comprised of the Directors of Mental Health at ten medical centers. These ten medical centers offer both inpatient and outpatient care, provided by more than 1,000 mental health clinicians of various disciplines [4]. There is considerable variation in the organization of the ten mental health services. The SC MIRECC functions as a 'virtual' center within VISN 16 and is charged with developing new knowledge about mental illness and its treatment, and with bringing new knowledge to bear on routine clinical care. The SC MIRECC works closely with the network mental health manager and the advisory council of mental health directors to identify the primary training needs of clinicians in the network and to implement training programs. Each of the training programs described here was developed in response to requests by the advisory council of mental health directors.

### Group therapy skills training

In 2001, faced with increasing demand for mental health services coupled with little expansion in the number of available mental health providers, the advisory council asked the SC MIRECC to provide an educational intervention to train providers in group therapy skills and techniques. A substantial amount of literature attests to the efficacy and effectiveness of group treatment modalities [5,6], yet an assessment of 136 mental health providers in VISN 16 indicated that only about one-third had any training at all in group therapy methods. We naïvely approached this training with the notion that an adequate learning experience was key. We gave little attention to implementation issues. Because the mental health leadership across the VISN had specifically requested this training, we assumed that local mental health programs would be invested in implementing the training.

### Intervention design and content

The group therapy skills intervention delivered a two and a half hour video conference on group therapy to mental health clinicians at 10 VA medical centers in the network. Clinicians at five of those medical centers (designated as our intervention sites) received additional extended training consisting of an intensive two and a half day face-to-face workshop, and eight monthly supervision conference calls provided by the trainers. Trainings were based upon a curriculum developed by the American Group Psychotherapy Association (AGPA) and were delivered by two certified and experienced group therapists/trainers from the AGPA program. In order to assess intervention effectiveness, we designed the intervention to allow us to compare the results of the extended training intervention with a control group receiving only brief training. To identify intervention and control sites, we examined key characteristics of the ten participating network facilities, including size of facility, number of patients, number of providers, etc., and created five matched pairs of facilities. Within each pair, an intervention site and a control site were randomly designated.

### Trainees

Clinicians who participated in the group therapy training were selected by their local mental health director with no input from the SC MIRECC. Clinicians (n = 136) located across the ten medical centers watched the two and a half hour videoconference and 36 clinicians at five sites received the more intensive training. Of the clinicians who received the intensive training, 8.3% (n = 3) were physicians, 13.9% (n = 5) were psychologists, 38.9% (n = 14) were social workers, 30.6% (n = 11) were nurses, and 8.3% (n = 3) were a psychological technician, a physician assistant, and an occupational therapist. In a post-training assessment, most trainees expressed satisfaction with the training experience and stated that they planned to initiate new groups in the next six months.

### Outcomes – initial training

To assess the extent to which the group therapy training was actually implemented in practice, we used VA administrative data to examine changes in the percentage of outpatients receiving group treatment at each site, and the percentage of outpatient visits that were conducted using a group format at each site. Group treatment was defined as psychoeducational, medication management, process, and psychotherapy groups in general VA outpatient mental health care clinics. PTSD and substance abuse programs were excluded because they typically provide treatment in group formats. Changes in these measures were studied at baseline (the three-month period prior to training) and again at 12 months. We examined these outcome measures in two ways, comparing results in each pair of five facilities and comparing the five control sites to the five intervention sites. We found no significant differences in any of the pair-wise comparisons. Only two sites (one control and one intervention site) demonstrated increases in both outcome measures at 12 months; the remaining sites had an unsystematic pattern of findings. We concluded that the training had not resulted in increased use of group treatments. As our initial aim was to increase the amount of group treatments being delivered, we did not assess the quality of group treatments that were delivered.

To understand why the training did not change patterns of care, we evaluated key informant interviews that we conducted post-training with 18 (50%) of the individuals who had participated in the more intensive training program. While most respondents stated that they valued the training, they also expressed a desire for greater structure and guidance in implementation and use of the follow-up consultation calls. Trainees at one site felt that they were forced to participate in the training but were not interested in doing so. Trainees identified several facilitators and barriers to starting a new group during the 12-month follow-up period, including administrative encouragement and mandates. Barriers included lack of time to plan and conduct additional group therapy sessions; practical limitations, such as lack of space; patient hesitation to participate in group therapy; and the lack of trained co-therapists to assist with the groups.

Although trainees rated the group therapy training highly, our evaluation suggested that simply providing training and ongoing supervision, even when identified as a priority by local administrators, was not enough to actually translate clinical training into everyday care. Even though the SC MIRECC had invested considerable resources in the training, practice patterns had not changed, and it is likely that few patients benefited. We concluded that future training interventions would need more attention to, and facilitation of, both training and implementation [7,8]. That is, training must be tailored to the trainees, and both trainees and administrators must be actively engaged in adoption of the new practice [3].

### Psychosocial rehabilitation training

In 2003, as the VA began to emphasize a recovery-oriented model of care [9], the network's mental health leadership requested that the SC MIRECC coordinate and deliver a training program in psychosocial rehabilitation techniques. A survey of the ten mental health services found that while limited rehabilitation services were offered at the two largest medical centers, none of the ten medical facilities provided a comprehensive psychosocial rehabilitation program. No network mental health clinicians had received formal training in psychosocial rehabilitation skills. We designed this next training effort with the lessons of the group therapy training in mind. We concluded that part of our role as deliverers of training was to supply the facilitation needed to translate the training to real world application.

### Intervention design and content

Because the need for training seemed clinically urgent across our network, we elected to offer training across the network and to use a simple pre-post design with no control group. The SC MIRECC training facilitator asked trainees and their site mental health services directors to create concrete and measurable goals for putting the training into practice, and we planned to assess the success of the training by the extent to which each individual site met its goals. One of the authors (MK) served as the SC MIRECC facilitator and assumed the responsibility of assisting with training and with local implementation issues [10].

The SC MIRECC contracted with the Center for Psychiatric Rehabilitation (CPR) to conduct psychosocial rehabilitation training. The CPR training usually consists of four semester-long courses at the University of Chicago and is a train-the-trainer model, based on 125 core competencies [11]. For our purposes, the CPR shortened the face-to-face experiential portion of the intervention to five days and provided two experienced trainers for on-site training. However, prior to arriving at the training site, participants were required to read four books and several articles as background. Four months prior to the training, the SC MIRECC facilitator began monthly conference calls to discuss the trainees' progress in the preparatory reading, their understanding of content, and their personal goals for the training. At the completion of training, participants were required to pass a qualifying exam and demonstrate competency via videotaped role-plays. After the face-to-face training, the trainers conducted a six-month booster session via teleconference.

### Trainees

The mental health directors at nine facilities nominated several potential training candidates, with a few additional candidates nominated by other clinical leaders. An enthusiastic, knowledgeable trainee leader emerged who agreed to assist the SC MIRECC facilitator in screening nominees. From a pool of 22 nominees, the SC MIRECC facilitator and participant-leader identified 16 candidates whom they expected to excel in the training program. These included three psychologists, six social workers, five nurses, and two vocational rehabilitation specialists. Trainees' post-training evaluations of the training were uniformly high. All trainees earned their certification as trainers in the CPR program.

As noted above, the SC MIRECC facilitator required trainees to set personal implementation goals, meet with their mental health director to develop site-specific implementation goals prior to training, set the plan in motion post-training, and train at least five clinicians in at least one recovery module. Site goals reflected the facilities' unique needs and capacities. Examples of site goals included establishing case management, initiating a skills training group, and commencing a compensated work therapy program. Trainees submitted their site goals in the form of an action plan prior to the training. The nine participating mental health directors agreed to commit resources to support the training of clinicians at their facilities and to participate fully in the evaluation of the intervention. Resources included the cost of travel to the training and local support for implementation, such as release time for program development and staff training, clinical space, administrative support, and purchase of educational materials. In addition, the SC MIRECC facilitator joined monthly calls with the mental health directors for one year to call attention to the intervention, report on trainee activities, and remind directors of their commitment, such as adding or expanding psychosocial rehabilitation services.

### Outcomes – second training

To evaluate the success of the psychosocial rehabilitation training, we assessed the extent to which each site met site-specific goals. Data were collected using structured key informant interviews conducted by phone. At twelve months post-intervention, thirteen trainees and nine directors completed interviews. (Three trainees were unavailable or lost to staff turnover.) Eight of nine facilities not only met but exceeded – and in some cases far exceeded – their site goals of adding new services or modifying existing services. Eleven trainees identified new services that they expected to provide in the subsequent six months. Seven of the thirteen trainees noted that over time they had actually revised their initial personal goals to be more ambitious. Rather than the projected 80 additional clinicians trained, at one year post-intervention, more than 300 additional clinicians in the network were trained in at least one component of the CPR program.

In post-intervention qualitative interviews, participants reported that setting goals, developing implementation plans, receiving support from management, and participating in follow-up conference calls with trainers and the SC MIRECC facilitator all aided implementation efforts. Trainees noted that the monthly calls with the SC MIRECC facilitator were especially valuable for sharing information and problem-solving around implementation challenges. In addition, the SC MIRECC felt it was important that both trainees and administrators were involved in setting goals and planning for implementation. Rather than the SC MIRECC supporting the full cost of training and choosing uniform outcome goals, each site invested in the training and took ownership of their goals for training outcomes. Motivated trainees invested considerable time preparing for the training. Administrators dedicated travel dollars and other resources (*e.g.*, time with leadership, space) to implementation, and both clinicians and administrators collaborated to identify mutual goals and address site-specific barriers.

Even though the SC MIRECC facilitated implementation for 12 months post-intervention, the trainees elected to continue meeting monthly for an additional 18 months. In effect, they formed a kind of informal learning collaborative – a self-directed group focused on practice and systems changes [12]. At the request of this collaborative, the SC MIRECC facilitator served as a consultant and supported additional advanced training. Some of the trainees have been asked to consult with and provide training for clinical programs in other VA networks, and the group has developed a sense of themselves as 'experts'. In addition, the training positioned one of the network mental health services to apply for and receive a VA-funded psychosocial rehabilitation fellowship program.

### Lessons learned

To summarize what we gleaned from these two interventions, we use a framework based on Fixsen's model of implementation [13]. Fixsen's model was not available when we designed the second intervention. We have modified this model to emphasize two main components: facilitation of training and facilitation of implementation (see Table 1). We feel this implementation model is especially appropriate for educational interventions since it distinguishes between clinician training (skills transfer) and implementation of the skills in practice settings.

**Table 1:** Lessons Learned and Applied in Facilitating Training and Implementation

	**Lessons Learned and Applied**
**Facilitating Training**	

**Participant Selection**	• Selection of trainees should be based on motivation and interest
	• Where possible, trainees should have similar levels of baseline knowledge
	• Trainees should commit to some performance standards, as well as set individual goals
**Training Content and Process**	• Trainers should be familiar with trainees' work environment
	• Certification or a marketable skill should be offered to motivate trainees
**Consultation & Coaching **	• Individual goals should guide post-training activities
	• Post-training consultation should be targeted to concerns expressed by trainees
	• Regular coaching should emphasize initial goals and performance standards
	• Goals should be modified if necessary as the process unfolds

**Facilitating Implementation**	

**Evaluation Approach**	• Utilize objective assessment of skills acquisition
	• Concrete site-specific outcomes should be assessed
	• Multiple follow up goal assessments allow for real time feedback to the extent possible
**Administrative Support**	• Collaboration between administrators and clinicians should ensure mutually-identified goals
	• Administrators should agree to commit the resources necessary to accomplish goals
	• Each stakeholder should receive recognition for efforts from administrators
**Systems Intervention**	• All stakeholders should be engaged in implementation and monitoring
	• Training should support the organizational strategic plan

Based upon Fixsen et al. (2005) [13]

For facilitating training, we have grouped three of Fixsen's domains: participant selection, training content and process, and post-training consultation and coaching. Specific requirements for success in each domain are shown in Table 1, and address issues such as selection of trainees based on motivation and interest and offering certification as a way to motivate trainees.

For facilitating implementation, we included Fixsen's remaining three domains: evaluation approach, administrative support, and systems intervention. Specific recommendations for success in each of these domains are shown in Table 1, and address issues such as having all stakeholders engaged in implementation and monitoring and including multiple goal assessments as a process measure.

### Planning to test the value of external facilitation

In this second training experience, we noted that facilitation seemed especially helpful in both the training and implementation components of the intervention. We distinguished between internal facilitation (provided by local change agents) and external facilitation (provided by an outside expert who assists with implementation) [10]. Both internal and external facilitation seemed to play important roles in this successful intervention. Internal facilitation was provided by local administrators in that they developed proactive plans and contributed resources. Trainees, and particularly the trainee-leader, also provided internal facilitation in terms of developing a kind of informal learning collaborative and serving as local advocates or 'internal change agents'. The external facilitation offered at a distance via conference calls by the SC MIRECC facilitator seemed to be especially critical. The SC MIRECC facilitator assisted with organizing the pre-training and training phases, encouraged and provided resources for the learning collaborative and ongoing training, and also helped to solve local problems that emerged during implementation. This external facilitation was not terribly time-consuming for the SC MIRECC facilitator, and to the extent that it promoted successful implementation was likely to have been cost-effective. We will investigate the cost effectiveness of external facilitation in our third intervention.

Our third intervention will build upon the distinctions we have made between training and implementation components and between internal and external facilitation. For this effort we plan to provide cognitive behavioral therapy (CBT) training uniformly across the network and, again, will require that individual sites (trainees and administrators) create implementation goals. Trainers will provide face-to-face educational programs and offer post-training supervision. An external facilitator will encourage and support application of CBT and promote a supportive special interest group. We expect that ten VA medical centers and several community-based outpatient clinics will participate and from these sites we will identify matched pairs. One of each pair will receive external facilitation during the implementation phase while the other will not. We will then assess the extent to which each site meets its implementation goals, comparing both outcomes and cost for those sites with and without external facilitation. Our hypothesis is that external facilitation of CBT implementation will result in better clinical outcomes and will improve the cost-benefit ratio.

## Discussion

In the course of conducting two large-scale clinical training programs, we learned several valuable lessons that contributed to the success of the second intervention. Although these 'lessons' have been noted previously in the literature [2,3,8,10,14,15], our experience reinforces the importance of taking a comprehensive approach to adopting practice changes. Promoting change by intervening at multiple levels internally (e.g., the clinician trainee, the clinic administrator, the mental health service leadership) seems especially important [2] and utilizing external facilitators to assist at these multiple levels seems promising. Fixsen *et al. *provide a useful model for thinking about practice changes in complex systems, which fits with our experience [13].

Many interventions to facilitate the uptake of evidence-based practices into routine care place a great deal of emphasis on the training experience but devote little time or effort to promoting implementation. Our first intervention is an example of such a poorly conceived approach. As it is unlikely that many patients actually benefited from this intervention, we concluded that the resources were not wisely used. Since large-scale clinical training programs are expensive and resources are limited, attention to implementation is critical if we are to use these resources to truly benefit patients.

Implementation studies typically utilize designs that compare the success of a multi-faceted training and implementation effort to the 'treatment as usual' condition. Indeed, this has been the design used in the majority of large-scale effectiveness research to date. It may be time for us to open the 'black box' of implementation and begin to test the value of specific components of implementation, such as external facilitation, in both (or either) training and implementation phases [16]. While implementation researchers have asserted that external facilitation has been helpful in a range of translation projects [10,13], there has been no systematic examination of its value [17]. Since external facilitation appears to be a relatively low-cost strategy, it is possible that it is cost-effective.

## Limitations

Although we did evaluate the outcomes of our first two interventions, in the context of this paper we view them predominantly as pilot case studies for large-scale training interventions. It is often necessary to refrain from interpreting case study findings too positively [18]. Indeed, there were key differences between our first and second training interventions that might have been relevant to their success or failure. Group therapy was not being promoted nationally by the VA when our training was done, although group therapy was identified as a priority by the directors of mental health in VISN 16; whereas psychosocial rehabilitation was central to the VA's national effort to establish a recovery model of care. Implementing group therapy approaches to treatment could have been in some ways more challenging than implementing psychosocial rehabilitation programs. For example, group therapy represents an approach that is a change to, or substitute for, previous practice. As such, it may be resisted by both patients and providers who are accustomed to one-on-one therapy. In contrast, psychosocial rehabilitation was seen as a new type of service in most settings rather than as a modification of existing practice and thus may have received more enthusiastic support. These and other differences between the two trainings could have contributed to their success or failure. These issues must be taken into account when contrasting and comparing the two initial training efforts.

## Summary

Achieving effective educational interventions that reach patients in complex systems is challenging. To improve the quality of care, it is not enough to simply train. Innovations must reach the patient. Previous research suggests that many well-intentioned, but ultimately ineffective training methods fail because they neglect to address the complexities of implementation, particularly the unique needs and multi-level barriers at implementation sites. Our initial experience adds to this evidence. From that experience, we identified several important lessons that illustrate the comprehensive, multi-level approach required to implement practice changes. In particular, external facilitation of both training and implementation at the level of the clinician, clinic, and facility appeared to be critical to the success of our second training program. We are now poised with a third intervention to open the 'black box' of implementation and test the added value and the cost-effectiveness of facilitation. Our work contributes to the literature by demonstrating how implementation principles can be applied in real world settings and describing the potential value of external facilitation for making changes in complex systems.

## Competing interests

The authors declare that they have no competing interests.

## Authors' contributions

All authors read and approved the final manuscript. GS and MK conceived of the two studies, developed their design, and helped to write the manuscript. MK coordinated the two studies described here. DB led the evaluation of both studies, performed the statistical analyses, and drafted an earlier version of this paper.
